# Local Structural Modifications in Metallic Micropillars Induced by Plasma Focused Ion Beam Processing

**DOI:** 10.3390/ma16227220

**Published:** 2023-11-18

**Authors:** Kritika Singh, Surya Snata Rout, Christina Krywka, Anton Davydok

**Affiliations:** 1 Institute of Material Physics, Hemholtz-Zentrum Hereon, Outstation at DESY Notkestr 85, 22607 Hamburg, Germany; kritika.singh@hereon.de (K.S.); christina.krywka@hereon.de (C.K.); 2 School of Earth and Planetary Sciences, National Institute of Science Education and Research, HBNI, Jatani 752050, India; surya.rout@niser.ac.in; 3 Homi Bhabha National Institute, Training School Complex, Anushaktinagar, Mumbai 400094, India

**Keywords:** focused ion beam milling, TiAl, magnesium, synchrotron, nanodiffraction, X-ray fluorescence

## Abstract

A focused ion beam scanning electron microscope (FIB-SEM) is a powerful tool that is routinely used for scale imaging from the micro- to nanometer scales, micromachining, prototyping, and metrology. In spite of the significant capabilities of a FIB-SEM, there are inherent artefacts (e.g., structural defects, chemical interactions and phase changes, ion implantation, and material redeposition) that are produced due to the interaction of Ga^+^ or other types of ions (e.g., Xe^+^, Ar^+^, O^+^, etc.) with the sample. In this study, we analyzed lattice distortion and ion implantation and subsequent material redeposition in metallic micropillars which were prepared using plasma focus ion beam (PFIB) milling. We utilized non-destructive synchrotron techniques such as X-ray fluorescence (XRF) and X-ray nanodiffraction to examine the micropillars prepared using Xe^+^ ion energies of 10 keV and 30 keV. Our results demonstrate that higher Xe ion energy leads to higher density of implanted ions within the redeposited and milled material. The mixing of ions in the redeposited material significantly influences the lattice structure, causing deformation in regions with higher ion concentrations. Through an X-ray nanodiffraction analysis, we obtained numerical measurements of the strain fields induced in the regions, which revealed up to 0.2% lattice distortion in the ion bombardment direction.

## 1. Introduction

Focused ion beam (FIB) milling is a widely employed technique within the realm of materials science, specifically designed for nanoscale analyses and preparing micro- and nanoscale samples for various investigative techniques such as transmission electron microscopy (TEM), atom probe tomography (APT), and X-ray nanotomography [1,2,3]. Its significance becomes particularly pronounced regarding sample preparation accuracy for in situ and operando tests at the micro- and nanoscale. However, caution must be exercised, because FIB milling involves bombarding the material of interest with high-energy ions, which can induce surface alterations and structural defects, including recrystallization, amorphization, formation of new phases, and material redeposition [4,5]. Therefore, the alterations introduced by FIB milling can significantly impact the validity of experimental results.

Various critical factors, including the choice of ion species, beam energy, milling conditions, sample temperature, and the initial structure of a sample influence the implantation of ions during FIB milling. Ongoing research in this field has continually uncovered new insights and opportunities for process improvements. In conventional FIB milling, the interaction of Ga^+^ ions with the sample causes ion implantation and formation of defects and dislocations which lead to amorphization. The implanted Ga^+^ ions can form alloys or intermetallic phases (e.g., with Cu), segregate along grains boundaries (e.g., Al- and Au-based samples) [6], and lead to phase transformation due to the generation of heat. Finally, during ion milling there is significant redeposition of materials which leads to mixing of the implanted ions. It has become evident that the presence of Ga^+^ ions in FIB-milled small-scale structures can significantly impact the properties of the milled specimens. To mitigate some of the artefacts produced by the use of Ga^+^ ions, an inductively coupled Xe^+^ plasma ion source has recently been added to FIB-SEM systems and has shown promising results. Multiple comparative studies on Ga^+^ and Xe^+^ ion-induced damage have been conducted on diverse materials [7,8,9,10]. For instance, a study by Xiao et al. demonstrated the significance of Ga^+^ FIB. milling in sample preparation [11]. In their investigation, FIB machining preceded atom probe tomography, TEM studies, and mechanical testing. The enrichment of Ga^+^ ions at grain boundaries was found to be detrimental, especially for specimens with numerous high-angle grain boundaries in Al alloy. The Xe^+^ PFIB milling process has been shown to produce relatively clean surfaces with no enrichment detected at grain boundaries and a thinner amorphous layer in the alloy compared to the conventional Ga^+^ FIB process [11]. Three-dimensional tomography of bainitic reactor vessel steel and WC-Co hard metal demonstrated 60 times faster milling rates with low artefacts using the Xe^+^ PFIB process [12]. The surfaces of Xe^+^ PFIB-milled samples showed low damage/amorphization, which was exhibited by high indexing rates during electron backscattered diffraction (EBSD) studies of the surface. Atom probe tomography has been used to quantify the implantation depth of four FIB ion species, i.e., Xe, Ar, N, and O, with different accelerating voltages on pure tungsten [13]. Xe showed the lowest implantation depth among all ion species for all accelerating voltages. Most studies have investigated conventional FIB sample preparation protocols (TEM lamella and APT needle) and have focused on standard materials like Si, W, and Al. Another study directly compared the artefacts induced by Ga^+^ and Xe^+^ FIB milling on high-entropy alloy microstructures by evaluating the mechanical properties [14]. In this study, TEM investigations were complemented by in situ tensile straining tests. The results indicated that samples prepared using the Ga^+^ FIB process exhibited higher strength, but lower ductility compared to those prepared using the Xe^+^ FIB process, where ions induced smaller damage zones beneath the amorphous layer. Furthermore, the Xe^+^ FIB samples exhibited superior material removal rates compared to the Ga^+^ FIB samples, making it a preferable choice for submicron sample preparation.

There are various synchrotron-based studies that have addressed the issues of changes in samples due to FIB processing, for example, the utilization of X-ray Bragg ptychography for 3D tungsten nanostructure reconstruction after He implantation using the FIB process. The ion beam processing was found to create partially deformed regions around the sample, as well as to induce lattice strain and lattice rotations, and such effects were meticulously observed and described through high-quality and extensive 3D maps [15]. Another investigation, using Bragg coherent diffractive imaging (BCDI) on FIB-produced Au microstructures suggested that thermal annealing could potentially mitigate the undesired artefacts resulting from the milling process. The authors elaborated on the Ga^+^-induced defects in Au particles and their behavior under elevated temperatures [16].

In this work, we present synchrotron studies aimed at conducting a quantitative exploration of the changes arising from FIB milling on metallic micropillars. As indicated by the majority of the referenced studies, ion energy ranging from 5 to 30 keV is commonly employed for the initial milling stages in FIB sample preparation for TEM investigations. In our study, we specifically concentrated on samples subjected to coarse milling, anticipating a higher concentration of ions and more pronounced structural deformation. The sample sets were produced using ion energies of 10 and 30 keV, striking a suitable balance for the materials under investigation and the milling time, falling within the typical ion energy range for microstructure milling. Specifically, we focused on two types of metallic micropillars, i.e., TiAl [17,18] and Mg alloy [19,20]. Both materials have been subjected to intensive investigation at Helmholtz Zentrum Hereon, where FIB milling for sample preparation is routinely employed. In both cases, PFIB milling was utilized to prepare the samples for study. Our approach involved the use of X-ray fluorescence (XRF) with a hundreds of nanometer synchrotron beam size to locate ion implantation and material redeposition with Xe^+^ ions. We applied this method to TiAl micropillars prepared with different ion energies. For analysis of the Mg micropillars, we employed XRF to locate ions and scanning X-ray nanodiffraction to investigate local lattice structure changes. On the basis of the 2D maps recorded from the samples, we were able to directly correlate the presence of ions with lattice distortion. Additionally, our results offer numerical characterization of the presence of redeposited ions based on the energy utilized during the milling process.

## 2. Materials and Methods

The cylindrical micropillar samples were meticulously produced using a TESCAN (Dortmund, Germany) Amber X Plasma Focused Ion Beam-Scanning Electron Microscope equipped with an Xe^+^ ion source. The preparation involved milling each material with two ion energies, i.e.,10 keV and 30 keV, utilizing a milling current of 10 nA and maintaining an incident angle of 90°. Consistency was maintained in both voltage and current throughout the plasma focused ion beam (PFIB) preparation, ensuring reliable identification of the implantation depth for each specimen at their respective accelerating voltages.

The experimentation was performed at the Nanofocus Endstation of the P03 beamline at the PETRA III synchrotron radiation source at DESY (Hamburg, Germany) [21]. The photon energy was meticulously set at 12.98 keV through the adept manipulation of a double crystal monochromator. A noteworthy aspect of the study was the refinement of the X-ray beam, which ultimately determined the experimental resolution. This precision was achieved by narrowing the beam’s dimensions to 250 × 350 nm^2^ (H × V) through the use of a sophisticated KB mirror system. X-ray fluorescence (XRF) spectra were captured utilizing an Amptek 123 XRF detector with 2048 channels which was strategically positioned at a distance of 30 mm downstream from the specimen. Simultaneously, the X-ray nanodiffraction signals were diligently recorded using a DECTRIS (Baden, Switzerland) Eiger 9M detector. The detector featured a pixel size of 75 × 75 μm^2^ and was positioned 192 mm away from the specimen, ensuring comprehensive data capture. Crucial to the precision of the measurements was the calibration process, undertaken with meticulous care. The photon energy and the sample-detector distance were judiciously calibrated, drawing upon the reliability of standard powder LaB_6_ as a reference.

## 3. Results

**Figure 1:**
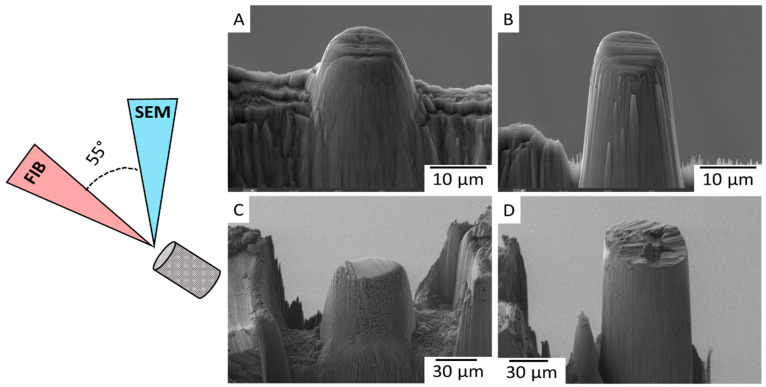
Schematic arrangement of the FIB-SEM column with micrographs of the TiAl micropillars milled at (**A**) 10 keV and (**B**) 30 keV, and the Mg micropillars milled at (**C**) 10 keV and (**D**) 30 keV.

In the studies presented, all four micropillars were fabricated with identical milling geometry. The materials under investigation were subjected to ion bombardment along the normal to the surface, while the electron beam for SEM imaging was directed at a 55° angle, as schematically illustrated in the inset of Figure 1. Following the protocol for coarse milling, utilizing 10 and 30 keV for each material, the micropillars of TiAl and Mg were prepared. Figure 1A,B show the TiAl micropillars after milling at 10 keV and 30 keV, respectively. The micropillar prepared with ion energy of 10 keV has a diameter of 15 μm and height of 13 μm, when the micropillar of 30 keV with similar diameter is 30 μm high. Initially intended for use in nanoindentation experiments, we conducted an inspection of the micropillars for redeposited ions before proceeding with fine milling and final preparation. Figure 1C,D depict Mg micropillars prepared using two different ion energies, i.e., with 10 keV and with 30 keV, respectively. In this instance, we once more observed a relatively short micropillar measuring 47 µm with a similar diameter, and a 70 µm high micropillar prepared with 30 keV ion energy, possessing the same diameter as the 10 keV micropillar at 47 µm.

**Figure 2:**
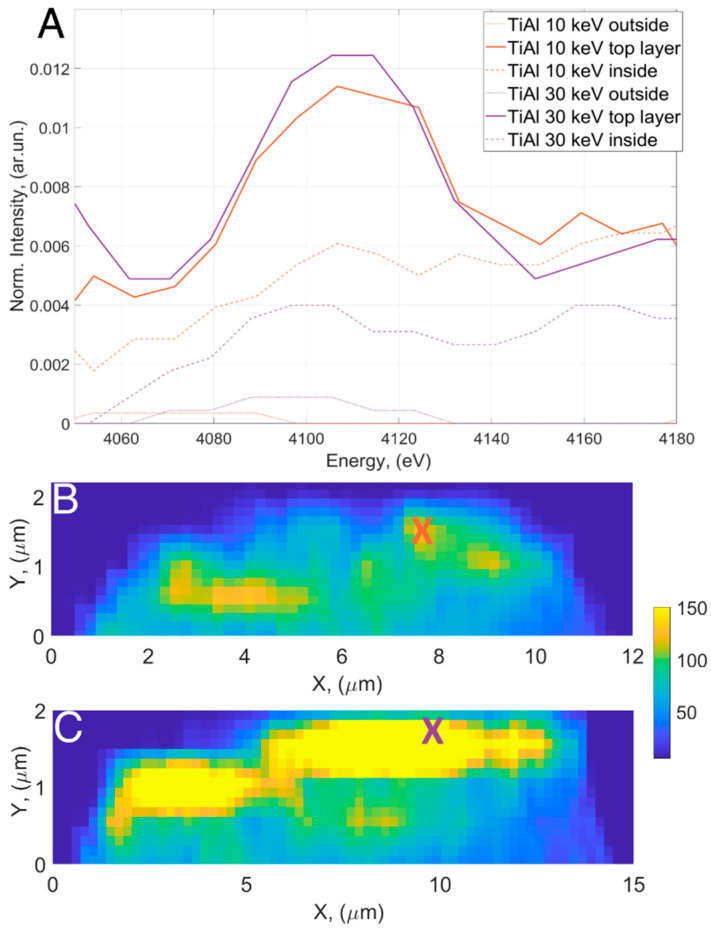
(**A**) XRF spectrum region at the Xe Lα1 line measured at three locations, i.e., outside the micropillar, high Xe content location, and 10 μm deep in the micropillars, from two TiAl micropillars prepared with ion energies 10 and 30 keV; (**B**) raster maps of integrated XRF intensity prepared from the region of interest (ROI) shown in micropillars prepared using 10 keV and (**C**) 30 keV Xe^+^ ions.

In this study, the X-ray fluorescence spectrum was limited by the sensitivity of the detector, which could only detect X-rays from 2 keV on one side, and the energy of the synchrotron beam, which was 12.98 keV, on the other side. The xenon emission Lα_1_ line is at 4109.9 eV and falls within this range. The line was used to identify the presence of Xe^+^ ions. Figure 2A shows a comparison of the XRF spectra within the region of interest (from 4000 to 4200 eV) from different locations on the micropillars. The signal from the region outside of the micropillar (air) was negligible, the spectra taken from a depth of 10 μm from the sample surface showed a signal at the same level as the background noise, while the spectra from the top layers exhibited clear peaks around the expected energy for Xe^+^ ions. By integrating the XRF region of interest shown in Figure 2A, two-dimensional distribution maps were created for both micropillars, as shown in Figure 2B,C. The top layer spectra shown in Figure 2A were recorded at the spot marked with an “X” on both maps. The maps indicate a concentration of Xe^+^ ions within the top 1.5 μm region of the micropillars, measured with synchrotron beam size precision. The concentration of ions, which determines the XRF intensity, differs depending on the ion energies used during preparation; higher XRF intensity corresponds to higher ion energy.

We also conducted XRF raster scans on the two magnesium micropillars prepared using ion energies 10 and 30 keV. During the Mg micropillar scans, a large area X-ray detector was used in the transmission geometry to record the nanodiffraction signal from each spot of the raster scans. The resulting X-ray diffraction patterns revealed reflections corresponding to the crystallographic planes (100), (002), and (101) of the hexagonal magnesium lattice. The micropillars under investigation were composed of a magnesium alloy, containing up to 10% gadolinium (Gd). The chemical composition influenced the lattice parameters, leading to observed values of a = 3.196 Å and c = 5.193 Å, which were derived from our experimental data and deviated slightly from the standard lattice parameters a = 3.20 Å and c = 5.21 Å for pure magnesium [22]. The reciprocal coordinates of the observed reflections were calculated and utilized as references for the strain fields calculations.

**Figure 3:**
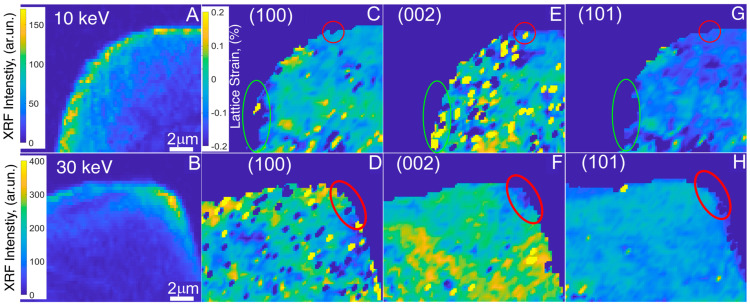
2D maps of Xe concentration in Mg micropillars prepared with (**A**) 10 keV ion energy and (**B**) 30 keV ion energy. Strain maps reconstructed for: The crystallographic direction (100), i.e., (**C**) 10 keV and (**D**) 30 keV; the crystallographic direction (002), i.e., (**E**) 10 keV and (**F**) 30 keV; the crystallographic direction (101), i.e., (**G**) for the 10 keV Mg–Gd micropillar and (**H**) for the 30 keV micropillar.

Figure 3 illustrates the two-dimensional graphs for both of the Mg samples. Panels C, E, and G represent the micropillar prepared with 10 keV ion energy, while panels D, F, and H correspond to the micropillar prepared with 30 keV ion energy. The graphs provide the strain distributions within the micropillars, shedding light on the effects of implanted ions during FIB milling on the crystal lattice. The xenon ion distribution maps, as shown in Figure 3A,B, and strain maps characterize the intricate effects of ion implantation on diverse crystallographic orientations. In the case of the magnesium micropillar prepared with 10 keV ions, distinct patterns emerge. A higher concentration of Xe^+^ ions can be observed in the lower left region and on the micropillar’s uppermost surface compared to the center of the micropillar.

Interestingly, the implanted Xe^+^ ions on the upper surface had caused localized compression along the (100) direction, highlighted by a red circle. Additionally, we observed localized expansion along the (002) direction and a relaxed state in the (101) direction (Figure 3E–G). Further down the micropillar’s sidewall, a more pronounced effect was noted, marked by green ellipses (Figure 3C,E,G). Similar effects were manifested in various directions, albeit with higher strain values, i.e., compression along the (100) direction up to 0.2%, expansion in the (002) direction up to 0.2%, and a slight compression in the (101) direction. In the case of the second micropillar milled at 30 keV, we noted a heightened concentration of ions, particularly clustered near the top edge of the micropillar (Figure 3B). Consequently, lattice distortion effects in this region were more significant and pronounced than that seen in the micropillar prepared at 10 keV. This specific region, where Xe^+^ ions were concentrated, is demarcated by a red ellipse on all three strain maps (Figure 3D,F,H). Within this marked area, we observed a slight expansion in the (100) direction, evident compression in the (002) direction, and a strain-relaxed region on the (101) map. Interestingly, the lattice distortions along the (100) and (002) orientations for both energies, i.e., 10 and 30 keV, are site specific. The lattice expansion along (100) and compression along (002) at the corner of the 30 keV-milled micropillar is a resultant of distortions observed along the sidewalls and top surface of the 10 keV Mg micropillar. The results and observations indicate that the most substantial damage due to ion implantation occurs in the direction of the ion bombardment, resulting in a slight expansion of lattice in the perpendicular direction. Moreover, the distortion is amplified with higher ion energies.

## 4. Discussion

**Figure 4:**
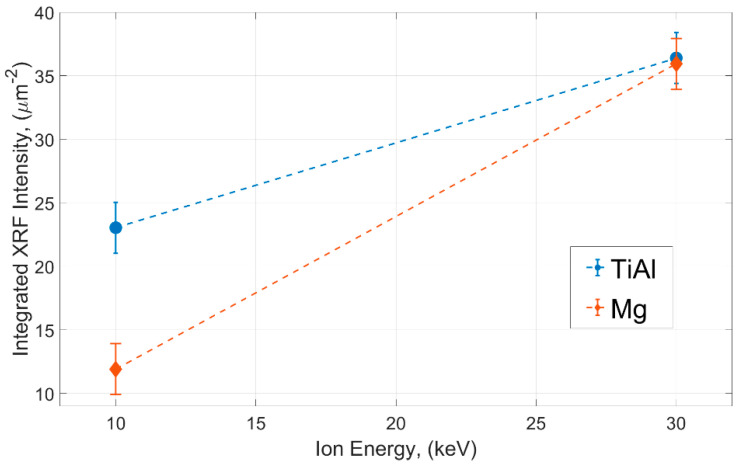
Variation of the integrated XRF intensity for the Xe Lα_1_ emission per unit area (µm^2^) as a function of ion energies for two materials TiAl (blue circles) and Mg (red diamonds).

Different methods have been used to characterize the phenomenon of ion implantation in FIB-processed specimens and in specific cases where proper sample preparation is essential for further studies. In the context of exploring new metallic alloys for different applications, the preparation of samples is a critical aspect that cannot be ignored, and it requires detailed and systematic investigation. Analyzing the effects of FIB milling on the structure is a complex task, especially following a secondary measurement. To address this challenge, we initially employed a synchrotron approach on samples prepared through coarse milling, where ion implantation and material redeposition is most prominent. Through this method, we were able to precisely locate implanted ions, study structural changes caused by the processing, and quantify ion concentrations based on the energy of the ions used (Figure 4). We described the concentration of redeposited ions per unit area by estimating ion intensities from XRF signals across the measured area, which varied with ion energy. The analysis provides insights into the behavior of materials under ion bombardment. At higher ion energies, the quantities of implanted ions are comparable for both TiAl and Mg. However, a notable contrast emerges at lower energies, i.e., the ion concentration for TiAl is twice as high as that in the case of the Mg micropillars. From this, we can infer that utilizing lower ion energy is advantageous for preparing Mg samples, as it substantially reduces the implantation of ions. Low-energy ion milling (<5 keV) is routinely used in TEM sample preparation to remove the amorphous layer that is produced during milling using 30 keV Ga^+^ ions. In addition, APT studies have shown very low Ga^+^ contamination of samples prepared using 10 keV Ga^+^ ions compared to those prepared using 30 keV ions.

Our study clearly shows that the removal of implanted Xe from the sidewalls of the micropillars by low-energy ion milling is dependent upon the density and Z of the atoms in the material. Distortion observed in the Mg micropillars is related to ion implantation and such intrinsic strain during FIB milling has already been observed in molecular dynamic (MD) simulations and X-ray diffraction studies [23,24,25]. During ion milling, stress levels up to 100 MPa can be reached, and the residual strain is dependent upon the energy and angle of incident ions. Within gold microcrystals, 30 keV and 5 keV Ga^+^ ions produce strain and defects within the top 50 nm and 20 nm, respectively, of the surface [24]. However, in this study, the Mg micropillars prepared using Xe^+^ ions showed strain concentrated within 1–2 µm of the surface or sidewalls. This is because of the longer tails associated with the focused Xe^+^ beam compared to the Ga^+^ beam at 10 nA. From the MD simulations, it is apparent that, during the initial stage of interaction of ions with a material, there is a temperature spike where the local temperatures can go above 1200 K [26]. This temperature spike along with the formation of defects leads to residual stress and the resulting strain in a sample. Recently, it has also been shown that Ga^+^ ion irradiation can lead to temperatures above 800 K in the irradiated region [27]. The temperature spikes in the sample during Xe^+^ irradiation should be considerably higher compared to those caused by Ga^+^ ions due to the heavier nature of Xe atoms. The higher temperatures along with high concentrations of defects (due to higher sputtering efficiency) of Xe^+^ ions should lead to formation of thick layers of amorphization and regions where strain in located. Significant care must be taken in choosing the appropriate energy and types of ions that are required to prepare samples using the FIB process. From our observations, even low energy Xe^+^ ions produce local strain within metallic samples. The concentration and level of strain will be much higher for nonmetallic samples.

Additionally, it is essential to consider the types of ions used during sample preparation. Most commonly used machines employ Ga^+^ or Xe^+^ ions, whose X-ray emission lines fall within the measurement range and sensitivity of XRF detectors. In contrast, ions like He, O, and N, which are occasionally used during FIB milling and have low X-ray emission lines, require a different XRF detector for detection.

### Angle-Dependent Implantation of Xe^+^ Ions

One of the important advantages of the PFIB process compared to the Ga-FIB process is the low preparation depth of Xe. However, the inductively coupled plasma source for Xe ions produces a larger focused beam diameter compared to a liquid metal source Ga-FIB. For example, the measured spot size for the 30 kV and 10 nA Xe beam used in preparation of the micropillars, in this study, has a diameter of 8–10 µm with large tails. Due to the presence of tails, the total effective region where Xe interacts with the sample is larger than 10 µm. The micropillars prepared from TiAl, in this study, have diameters in the range from 10 to 15 µm. While milling the side walls of the micropillars, the large spot size and tails lead to implantation of Xe ions on the top surface of the micropillars at an angle of 0° (angle calculated from the normal to the surface). Due to this, we see significant implantation of Xe ions on the top of the micropillars (Figure 2). But the micropillars from magnesium are larger in diameter and we see significantly less implantation of Xe ions on their top surface. On the sidewalls of micropillars the Xe ions should impact with glancing angles (80–90°) and implantation depths are lower [13,28,29] and radial. Implantation of Xe in the sidewalls should lead to amorphization as seen in lamellas prepared for TEM studies [10,13,28,29]. It has been shown that the thickness of amorphization for samples prepared with Xe ions is ~40% less compared to those prepared using a Ga-beam [10]. Such a comparative study will be done in the future for micropillars prepared using Ga and Xe ions. The low implantation in the sidewalls is clearly exhibited by the low XRF signal from the sidewalls compared to the top of the micropillars. The high Xe signal from the sidewalls of the Mg micropillars is predominantly due to redeposition of materials. Redeposition is an important and unavoidable effect during coarse milling procedures in a FIB and can be reduced by using low-kV beam polishing. Since our samples are not polished using a low-kV beam, the total implanted Xe in the sidewalls of all the micropillars should include contributions both from direct implantation of Xe and material redeposition. Due to the shape of the focused Xe beam with wide tails, the sidewalls are not homogeneously milled, and in the end, the micropillars have a wide base in the shape of a trapezium, and therefore, due to such effects, it is imperative to polish the samples with low current beams.

## 5. Conclusions

In conclusion, we have demonstrated a technique for mapping the implanted Xe^+^ ions resulting from the coarse FIB milling process and for characterizing the local structural alterations caused by this process in TiAl and Mg micropillars. By selecting a region of interest within the XRF spectrum centered around the Xe Lα_1_ line, we were able to reconstruct a 2D map illustrating the location of implanted ions. In the case of Mg micropillars, a structural analysis based on nanodiffraction data was conducted, revealing lattice compression in the direction of ion bombardment of up to 0.2% and simultaneous lattice expansion in the perpendicular direction, also at a magnitude of 0.2%. We calculated the amounts of implanted ions per unit volume through the X-ray technique. Our future plans involve populating the graph with data from other materials subjected to similar processing and varying ion energies; such investigations are expected to enhance existing FIB preparation protocols and to facilitate further explorations in this field.

## Data Availability

The datasets used and/or analyzed during the current study are available from the corresponding author on reasonable request.
